# Reducing the global burden of cancer

**DOI:** 10.1016/j.apjon.2022.100087

**Published:** 2022-05-27

**Authors:** Margaret I. Fitch

**Affiliations:** Bloomberg Faculty of Nursing, University of Toronto, Ontario M4C 4V9, Canada

## The cancer burden is increasing

The burden of cancer is rising around the world. In 2012, there were 14 million new cases of cancer diagnosed and 8 million deaths from the disease globally; these numbers are expected to increase to 22 million new cases and 13 million deaths annually by 2030.^1^ The increase is due to aging populations, and economic, societal and lifestyle changes. There is a strong correlation among socioeconomic level, human development, and cancer.^1^

The global burden of cancer is not rising in a uniform manner.^1^^,^^2^ Given the globalization of economics and lifestyle behaviors is not happening at similar rates, the result is uneven transitions in exposure to causative factors. Cancer is the leading cause of death in many high-income countries and may soon be in middle- and low-income countries as well. Presently, the incidence of cancer is higher in high-income settings, but mortality rates are higher in middle- and low-income settings. Three-quarters of the predicted increase in cancer burden is expected to occur in middle- and low-income countries, locations that are least prepared to handle this challenge.

## The cancer burden is broad

The rising incidence of cancer creates demands for access to diagnosis and treatment facilities. But cancer and its treatment create more than a demand for medical, surgical, and radiation services. Cancer and its treatment have impacts on individuals, families, and societies. There are not only physical impacts but also emotional, social, psychological, spiritual, informational, and practical challenges. It is well documented any of these issues can have a profound influence on the quality of life and eventual cancer related outcomes.^3^ One of the pressing emerging “toxicities,” which is now being recognized for many patients and families is financial. Having to pay for cancer care and medications to manage symptoms and side effects can cripple an individual and family and have an impact long after treatment is finished.

As more effective treatments are being implemented, larger proportions of individuals diagnosed with cancer are living longer than in the past, and these numbers are also expected to continue to grow, exceeding 20 million worldwide by 2025.^4^ Many of these survivors continue to live with ongoing consequences of the disease and its treatment and their experience has been likened to living with a chronic illness. Yet, these individuals often experience gaps in their follow-up care. In many countries, survivorship is not yet embraced as part of the cancer continuum.

Finally, in countries where the largest proportion of diagnoses are made at a late stage of the disease, palliative care must be a priority. Despite palliative care being declared a human right,^5^ many countries still do not have adequate access to this service, and many individuals are dying in pain and distress. Access to even basic pain management remains a pressing challenge in many locations faced with attitudinal, educational, and policy barriers.

## Effective strategies are needed

The reality of the increasing cancer burden is driving advocacy efforts and explorations to find strategies that would effectively reduce this challenge and mitigate the impact on individuals, families, health care systems, and societies. The strategies are needed across the cancer continuum beginning with primary prevention and also embracing strategies for secondary and tertiary prevention.

A key control strategy to reduce the incidence of cancer is primary prevention or the delivery of programs to populations that would reduce the exposure (potential risk) to factors we know cause the disease. If we were to focus our efforts on the factors we know can be (or potentially are) modifiable, it would increase the likelihood of changing some of the causal influences.^1^ Currently, based on what we know regarding the causes of cancer, one-third to one-half of all cancers could be prevented.

Modifiable risk factors of cancer include tobacco smoke, alcohol, overweight/obesity, insufficient physical activity, solar ultraviolet (UV) radiation, and dietary factors (ie, insufficient fruit, nonstarchy vegetables, and fiber; high intake of processed/red meat; high sat intake). Additionally, there are occupational and infectious causes of cancer, which vary from country to country. These factors contribute in different degrees to different types of cancer, resulting in variation in the types of cancer predominating in different countries. High-income countries have high rates of breast, prostate, colorectal, lung, and endometrial cancers, while low-income countries have high rates of prostate, lung, esophagus, cervical, and breast cancers. The variation has implications for which strategies may be required to raise awareness about the disease in the general population and the focus needed for prevention programs. How to effectively reach target populations and change the necessary lifestyle behavior(s) must be top priorities for prevention research and program development.

Although primary prevention strategies are key to reducing the burden of cancer and are likely the most successful and cost-effective approaches for countries to use, secondary and tertiary prevention strategies are also required to ensure early diagnosis and timely access to treatment and mitigate the impacts on the quality of life; barriers to early detection have been identified in many settings and often include person-specific, health care system and societal factors. Finances, attitudes, culture, infrastructure, policy, and societal norms can have important roles in shaping behaviors regarding screening for cancer and attendance at health care facilities.

Cancer care is considered a specialty practice requiring additional preparation for all health care professionals beyond their basic preparation. Although designation for cancer care as a specialty has been widely accepted in medicine, surgery, pathology, and radiology disciplines, this has not occurred in many countries for other members of the health care team. For example, nursing care or psychosocial care of cancer patients and their families is not considered a specialty in many countries, and access to the necessary specialty knowledge is limited. Additionally, there are projected shortages of all health care human resources around the globe, which will likely increase the challenge of developing specialty practitioners for cancer care.

Consideration of palliative care is also needed if the burden of cancer is to be reduced. Advocates of palliative care have emphasized that all health care professionals require general education in palliative care, while some require special education. A key strategy, as well, is developing programs whereby patients have ready access to opioids for pain management. In countries where such programming has been successful, multipronged strategies had to be implemented.

## Nurses have a key role in reducing the cancer burden

Nurses have key roles in cancer control and in the delivery of interventions that could reduce the burden of cancer. The very nature of their frontline care positions, their immediacy of contact with the public, the trust patients and families place in them, and the scope of their practice offers clear opportunities for influencing the cancer burden. However, it will require concerted efforts in advocacy, policy change, and leadership to fully realize this contribution. The recently released WHO Strategic Directions for Nursing^6^ recommends necessary strategies for human resource development and work environment change, which countries could implement to have nurses able to work to the full scope of their preparation. Such directions are critical for the overall practice of nurses and to have a foundation on which to build specialty practice.

Whether working in a general nursing role, specialty other than cancer, or in cancer care itself, nursing practice embraces elements of primary, secondary, and tertiary prevention. Given the widespread existence of cancer and the importance of primary prevention, all nurses need a certain level of knowledge about the disease. For example, teaching patients about risk factors for cancer, healthy lifestyle practices, and early warning signs ought to be part of every nurse’s practice. Being able to identify when a symptom could reflect a need for further investigation regarding cancer also ought to be incorporated by every nurse.

In terms of secondary and tertiary prevention, specialty education and the subsequent development of leaders, faculty, and researchers in the field, are critical. Building the capacity for specialty practice in cancer nursing care and palliative care is not only about developing practitioners, but it is also about enlarging the evidence base for practice and developing effective leaders who embody the vision and are able to influence policy change.

## Conclusions

Globally reducing the burden of cancer is not only about reducing the number of new cancer cases, although this is a critically important outcome. It is also about achieving several other outcomes. It is about helping individuals access early diagnosis and timely treatment. It is about working to have individuals and families who are knowledgeable about the impact of cancer treatment and capable of managing symptoms and side effects. It is about ensuring that individuals do not unduly suffer as they are dying. It is about having cancer survivors live to the fullest of their capabilities.

Nurses can play key roles in achieving these cancer control outcomes. But concerted efforts are needed regarding leadership, policy, education, and working environments to support nurses across the world and realize their potential for influencing cancer control.

## Funding

Nil.

## Declaration of competing interest

None declared.
